# Moving towards an enhanced community palliative support service (EnComPaSS): protocol for a mixed method study

**DOI:** 10.1186/s12904-015-0012-4

**Published:** 2015-04-30

**Authors:** Steven M Arris, Deborah A Fitzsimmons, Susan Mawson

**Affiliations:** School of Health and Related Research, University of Sheffield, Regent Court, 30 Regent Street, Sheffield, South Yorkshire S1 4DA UK; School of Health Studies, Western University, London, ON N6A 3B4 Canada

**Keywords:** Palliative care, Information and communication technologies, Mixed methods

## Abstract

**Background:**

The challenge of an ageing population and consequential increase of long term conditions means that the number of people requiring palliative care services is set to increase. One UK hospice is introducing new information and communication technologies to support the redesign of their community services; improve experiences of existing patients; and allow efficient and effective provision of their service to more people. Community Palliative Care Nurses employed by the hospice will be equipped with a mobile platform to improve communication, enable accurate and efficient collection of clinical data at the bedside, and provide access to clinical records at the point of care through an online digital nursing dashboard. It is believed that this will ensure safer clinical interventions, enable delegated specialist care deployment, support the clinical audit of patient care and improve patient safety and patient/carer experience.

Despite current attempts to evaluate the implementation of such technology into end of life care pathways, there is still limited evidence supporting the notion that this can be sustained within services and implemented to scale. This study presents an opportunity to carry out a longitudinal evaluation of the implementation of innovative technology to provide evidence for designing more efficient and effective community palliative care services.

**Methods:**

A mixed methods approach will be used to understand a wide range of organisational, economic, and patient-level factors. The first stage of the project will involve the development of an organisational model incorporating proposed changes resulting from the introduction of new novel mobile technologies. This model will guide stage two, which will consist of gathering and analysing primary evidence. Data will be collected using interviews, focus groups, observation, routinely collected data and documents.

**Discussion:**

The implementation of this new approach to community-based palliative care delivery will require significant changes to established working patterns. This new service delivery model is being developed by the Hospice in collaboration with a team of international academic, industry, and clinical commissioning service improvement specialists. The findings from this initial evaluation will provide valuable baseline evidence regarding the delivery of palliative and end-of-life care services.

## Background

Demographic shift, and the requirement to provide care for an increasing aging population, is a pressure point on global health systems that is yet to be addressed [1]. This is particularly true when considering palliative care for a burgeoning population with chronic health conditions [2]. There is extensive evidence regarding the benefits of palliative care to optimise patient quality of life [3-5]. However, services are frequently fragmented, and there appears to be a disparity of care provision, with patients with non-malignant disease either receiving palliative care in the very late stages of their illness, or not at all [6-8]. Whilst there is evidence that most people would prefer to die at home [9], a lack of specialist palliative support can lead to uncontrolled symptoms, including pain management [10], and the need to use out of hours or emergency medical services [11,12]. Consequently, many patients are admitted to hospital and are unable to die in the place of their choosing [13-16].

There is a significant need to prevent avoidable admissions to acute care settings for people nearing the end of their life. A recent survey of 200 sets of patient case notes in Sheffield, UK identified possible alternatives to hospital-based end of life care for 40 per cent of cases [15]. The potential savings in acute bed expenditure is £4.5 M per annum, equivalent to a national saving of £450 M. A similar, smaller, study in East Kent [16] indicated that 52% of admissions might have been avoided. This presents opportunities for people to die with dignity in the place of their choosing, in familiar surroundings and to make efficiency savings across the health economy. It also creates a challenge for effective palliative care delivery as it has been shown that family perception of the benefits and services received is dependent upon the consultation and support service delivery being located within the same health care domain [17]. Supporting more palliative patients in their own homes will consequently place even greater strain on already stressed community-based resources.

The English End of Life Care Strategy [18] identifies that palliative care in England is still largely regarded as “an optional extra”, and that the NHS relies heavily upon charities, including hospices, to deliver specialist palliative care. The aim of the strategy [19] was to bring about a step change in access to high quality care for all people approaching the end of life irrespective of their location, age, gender, ethnicity, religious belief, disability, sexual orientation, diagnosis or socioeconomic status. However, delivery of an effective and efficient high quality palliative care service continues to be a challenge. Following our own extensive stakeholder engagement, our community partners have identified that finding a way to provide high quality, yet affordable, palliative care in the community is of key importance to them.

The Palliative and end of life care Priority Setting Partnership has recently published a report [19] drawn from extensive public and practitioner engagement, identifying high priority research questions regarding the delivery of palliative care. Some of the top ten questions identified include:

1. What are the best ways of providing palliative care outside of working hours to avoid crises and help patients to stay in their place of choice;
2. How can access to palliative care services be improved for everyone regardless of where they are in the UK;
3. How can it be ensured that staff, including healthcare assistants, are adequately trained to deliver palliative care, no matter where the care is being delivered; and
4. What are the benefits, and best ways, of providing care in the patient’s home and how can home care be maintained as long as possible.

This project will be a first step in answering these questions by examining how an internationally proven model of care can be used to effectively address a significant gap in community-based palliative care in England, reducing needless and unwanted hospital admissions, leveraging scarce specialist palliative care resources, improving care decision-making and delivery, and extending service capacity.

A validated international system of information and communications technology (ICT) will be contextualised to the NHS. It will then be applied as an enabling mechanism to the delivery of community-based palliative care. This will enable one UK Hospice to transition from their current paper-based clinical information system when in the field to a real-time, mobile, digital-enabled system of patient clinical data capture, access, review and sharing. This will have a dramatic impact upon the delivery of care within the community by the palliative care specialists employed by the Hospice. Subsequently, the Hospice plans to leverage the new capabilities supported by this system and deliver a number of innovative palliative care delivery programs as part of their Enhanced Community Palliative support Service (EnComPaSS), including delegated care acts.

The objective of this paper is to present the protocol for the baseline study, including a detailed description of the study design and methodological approach. The methodology described in this paper will also act as a reference for future publications about this, and subsequent studies.

## Methods/design

### Study design

A mixed methods approach [20] based on a theoretical change model will be used to understand a wide range of organisational, economic, and patient-level factors, examine the barriers to, and facilitators of the implementation of this innovative service model [21], and to explain how and why specific changes are expected to come about [22]. The first stage of the project will involve the development of an organisational model incorporating proposed changes resulting from the introduction of new mobile technologies. This model will guide stage two, which will consist of gathering and analysing primary evidence to describe he actual changes achieved and explain any differences between these and the anticipated model. Whilst the theoretical change model will highlight areas where future developments are likely to create change, all outcomes of complex service reorganisation cannot accurately be predicted at the outset [23]. Consequently, a wide range of baseline data is required in order to provide comparisons in the case of emergent themes.

This initial study will:

1. Identify and document current working practices, including time spent on certain tasks, data management, and communication practices;
2. Identify and isolate the key costs of the service;
3. Establish current data input and data use, including data quality and resources required for data interactions (e.g. accessing patient notes, or producing service reports); and
4. Assess current patient outcomes.

Data will be collected using interviews, focus groups, observation, routinely collected data and documents.

### Setting

The study is based at St. Luke’s Hospice in Sheffield which serves a community of 551,000 [24]. The Hospice has a 20 bed inpatient centre, Therapies and Rehabilitation Centre, a community outreach service, and an educational service providing training in palliative care delivery for health care professionals.

### Intervention

The existing community outreach service was developed in line with best practice. The main features of the service are that:

- The service supports patients throughout their illness in their own homes;
- Each patient has their own designated community palliative specialist nurse to provide specialist knowledge, support and advice to them, their family and careers, and other health professionals caring for them;
- Working with General Practitioners, district nurses, hospital consultants, specialist hospital nurses and other health professionals, the service develops care programmers to meet the needs of each patient;
- The nurses provide patients and their families with emotional and psychological support;
- The service aims to ensure patients have the best possible quality of life, relief from symptoms, and flexible choices about where they are looked after;
- The nurses do not provide intensive home nursing or help with daily physical care, but if this is required, they can help to organise it through local care providers.

**Figure 1:**
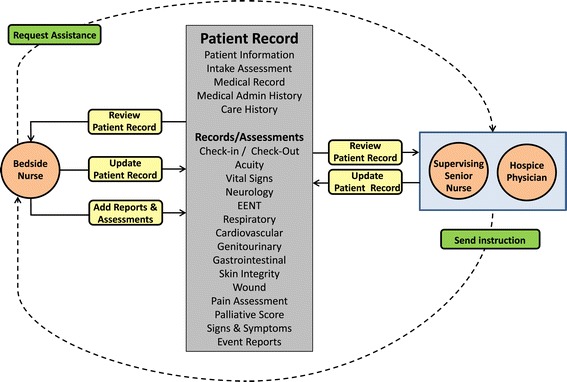
**Data flow.**

The nurses delivering the service in the community will now transition from a paper-based system to tablets loaded with software designed to capture patient clinical data at the bedside in an electronic format; display the data via an online dashboard; and improve communication, and the quality of shared information, across the service as shown in Figure 1. This highly innovative information and communications technology (ICT) will enable junior nurses, who would normally work in conjunction with a senior experienced nurse in the community, to work alone as the technology provides the nurse working at the bedside with the ability to request assistance online and in real-time from senior nurses and hospice physicians working within the hospice. The ICT also eliminates the need for nurses to return to the hospice to access or update digital care records, and will help improve the efficiency of multidisciplinary team meetings, thereby reducing travel effort and expense, supporting clinical decision-making at the point of care and enabling timely referrals, prescriptions and orders.

The digital capture of point of care data will provide current ‘at-a-glance’ clinical information reducing potential risk, such as product or dosage errors, and enabling immediate delegated practice and intervention from senior nursing and medical clinicians. Providing access to Patient Reported Outcome Measures (PROMs), the digital dashboard will also allow digitally-enabled observations management, such as risk profiling of patients, across the entire patient cohort and for all clinicians.

## Research methods

This study provides a unique opportunity to benchmark existing community palliative care delivery services with specialist care providers in preparation for a subsequent operational and financial evaluation of the new service delivery modality. Data will be collected using interviews, focus groups, observation, routinely collected data and documents.

### Observation

The evaluator will shadow staff members and record their activities with a combination of written notes and audio recording of discussions, this will subsequently be transcribed verbatim. The evaluator will not be present during the carrying out of care activities; they will remain in public spaces or staff areas and record activities related to work flows, organisational issues, record-keeping, communication and data management, rather than individual care of patients.

### Interviews/focus groups

Semi-structured interviews will focus on what is considered to work well, or not so well with current working practices, and on identifying how the future use of mobile technology could impact on service provision and patient experiences. This will support the identification of barriers to, and facilitators of the implementation of the new service model. Interview schedules will be developed in line with emergent findings from the observation stage. Focus group sessions will be conducted towards the end of the data collection phase to assess the face validity of emerging findings, and to explore areas of consensus and disagreement regarding descriptions of the current working model. Interviews and focus groups will be audio recorded and transcribed verbatim.

### Routine data

A wide variety of routinely collected data will be gathered retrospectively. These will contribute towards defining the service delivery model as well as the economic analysis, and will include staffing patterns, service costs, patient characteristics, referral routes and outcomes. When applicable, all data will be anonymized by hospice staff members before access to the data is gained by the evaluator. Data will be captured by the evaluator in Excel or SPSS spreadsheets for subsequent analysis.

#### Sampling, recruitment, access and consent procedures

The study population will be all staff members at the participating hospice involved in the delivery of the community palliative and end-of-life care services. A combination of purposive and convenience sampling will be used to identify potential participants.

### Observation

As the observation of working practices could involve interactions between individuals across the organisation, an opt-out method of general consent will be gained for these evaluation activities. All staff members will be advised by email that the observation will be taking place, and they will be asked to respond to the evaluator if they wish to be specifically excluded. The email will include a brief description of the observation activities. Staff members will be given a week to respond to this email, after which a list of the remaining staff members, categorised by job titles, will be used to purposively ensure that appropriate roles are represented in the planned observation sessions. Within these role categories, convenience sampling will take place depending on ease of access, shift patterns, types of activities being undertaken, availability of individuals etc. The convenience sampling will be organised through discussions with appropriate staff members. The short-list of potential participants will be approached and provided with a detailed information sheet, either by email or paper copy, and asked to respond to the evaluator if they wish to participate. Those that reply positively will then be contacted by the evaluator to arrange a suitable time for the observation sessions to proceed. The participants will receive an information sheet at least 24 hours prior to any observations being made, and will be provided with a paper copy of the information sheet and a consent form to be signed in duplicate prior to the commencement of the initial observation session. A phased recruitment process will be used to prevent over-subscription.

### Interviews

A list of the staff members, categorised by job titles, will be used to purposively ensure that appropriate roles are represented. Within these role categories, the advice of senior members of staff will be sought to decide on appropriateness of involvement (such as participation in community care, experience and use of data systems, knowledge of organisational processes etc.), an element of convenience sampling might take place depending on availability of individuals. Identified staff members will then be approached and invited to participate in either an interview through recruitment packs provided to them by email or paper copy. If they are willing to participate in the study, they will be asked to contact the evaluator to arrange a suitable time and place for the interview. It is most likely that this will be at the hospice premises, although if participants prefer, this can be arranged in a more convenient or desirable location. Participants will receive an information sheet at least 24 hours prior to attending the interview, and will be provided with a paper copy of the information sheet and consent forms when they attend the information session.

Whilst this method could potentially introduce an element of bias, (owing to resource and time constraints) only a small number of interviews will be carried out, and depth and appropriateness of data are considered more important than achieving broad representativeness of views at this stage.

### Focus groups

Two focus groups will be undertaken; one consisting of relatively senior members of hospice staff, and the other more junior members of hospice staff. The purpose of these focus groups is to check the face validity of emerging findings, to gain consensus and identify areas of disagreement about the current situation. The separation of senior and junior staff members will help to avoid response bias, which can result in co = present investigations owing to hierarchical relationships in organisations. Efforts will be made to recruit participants that have not taken part in the interviews or observations to redress any potential bias due to lack of representativeness in earlier stages. Identified staff members will be approached and invited to participate in a focus group through recruitment packs provided to them by email or paper copy. If they are willing to participate in the study, they will be asked to contact the evaluator to arrange for inclusion in the focus group. Participants will receive an information sheet at least 24 hours prior to attending the focus group, and will be provided with a paper copy of the information sheet and consent forms when they attend the focus group session.

Participants will only be recruited to the study if they can provide informed consent. The Department of Health [25] state that “for consent to be valid, it must be given voluntarily by an appropriately informed person who has the capacity to consent to the intervention in question”. To ensure that this is the case in this study, the following measures have been implemented.

### Voluntary consent

Department of Health guidance [25] states that for consent to be valid “it must be given voluntarily and freely, without pressure or undue influence being exerted upon them”. Every care will be taken to ensure that each participant is able to give voluntary consent and they will have the option to deny consent or withdraw from the research at any time without any negative consequences and without providing any reason or explanation.

### Timing of consent

Department of Health guidance [25] states that “it is good practice where possible to seek the person’s consent to the proposed procedure well in advance, when there is time to respond to the person’s questions and provide adequate information”. Consequently the researchers will provide potential participants with the information sheet which they can review at their leisure over a period of no less than 24 hours before a researcher contacts them and their consent to participate in the study is sought.

### Form of consent

Although completion of a consent form is in most cases not a legal requirement it is recognized as good practice, so written consent will always be obtained. Prior to their first observation session, interview or focus group, participants will be asked to complete two copies of a written consent form. One copy will be retained by the evaluator and the other by the participant.

This study will be undertaken over a five month period. The study will collect baseline data regarding the current operating practices for the St Luke’s Hospice community palliative care services, prior to the introduction of new technologies and changed ways of working.

### Data analysis

#### Observation

The notes and transcripts of audio data gained from the observations will be entered into NVIVO (v10). The purpose of the analysis will be both descriptive and interpretative [26]. Data will be coded according to these two priorities. The descriptive analysis will outline current working practices, focusing on data flows, communication, and organisational issues. The interpretative element will concentrate on assessments of the functionality of current working practices, and how the introduction of mobile technology and associated changes in working practices might be beneficial or detrimental.

#### Interviews

The interview transcripts will be entered into NVIVO (v10) and analysed thematically, based on the coding scheme developed during the analysis of observational data, further issues will be explored and the coding scheme will be refined and further themes/sub-themes added as they emerge.

#### Focus groups

Focus group transcripts will be entered in NVIVO (v10) and analysed according to a coding framework following the topic guide. Outputs will therefore reflect validity and consensus issues related to the emerging findings. Informed by the coding scheme from the interview analysis, potential further themes will be identified and added. Findings will provide evidence for the validity of the emerging service provision and theory of change models, and possible explanations within the data for levels of consensus will be investigated.

#### Routine data

Routinely collected data will be used to provide descriptive baseline statistics, financial and economic evidence for the service. Analysis will be managed in Microsoft Excel or SPSS when this is the most appropriate approach for the type of data and outputs required. Where the quantity and quality of the data allow, the analyses and descriptions will follow the templates created for service-level reporting of baseline data in community services in a previous study [27].

#### Data management and storage

All hospice staff data will be given an identification code, and a key to participants' identities will be stored separately and securely. Transcripts and notes will be anonymised when they are transcribed. When the context or specificity of role may identify a participant, this information will be omitted from any outputs, or more generic terms used in its place. However, in instances where potentially identifying information might significantly add to the accuracy and strength of findings, express permission will be sought from that individual to reproduce their data. All patient data will be aggregated and only be used to explore service level issues such as gender mix and service throughput, and will be anonymised at source.

The evaluator will act as custodian of the data. Data will either be captured on, or stored as soon as possible on encrypted devices. Audio data and hand-written notes will be destroyed once transcribed and transcripts identified with a code. Textual data will be stored on encrypted devices or password protected drives at the University of Sheffield. Should there be a need to share data with international members of the research team, data will be sent as an email attachment encrypted with a password, the password will be sent separately, and the data will be stored on a password protected drive within Western University.

#### Ethical approval

A favourable ethical opinion for this research proposal has been received from The University of Sheffield Research Ethics Committee (reference: 002550).

## Discussion

The implementation of this new approach to community-based palliative care delivery will require significant changes to established working patterns. The Hospice is currently collaborating with a team of service improvement specialists from the University of Sheffield, Western University (Canada), Sensory Technologies UK Ltd, the National Institute for Health Research (NIHR) Collaboration for Leadership in Applied Health Research and Care, Yorkshire and Humber (CLAHRC YH), and Sheffield Clinical Commissioning Groups (CCGs). The findings from this study will provide valuable baseline evidence for subsequent research regarding the efficiency, clinical and cost effectiveness of technology-enabled palliative care and end-of-life services, and the organisational changes required to achieve them.

### Strengths

A key strength of this study lies in the qualitative and quantitative mixed method approach which will enable rich exploration of the existing community-based palliative care service prior to the implementation of the new ICT enabled care modality. The ethnographic approach used will provide the research team with greater understanding of the current organisational processes and information flows, and will enable very detailed evaluation of the subsequent implementation. Engagement with a broad representation of the Hospice staff in the observations, interviews and focus groups will ensure their perspectives contribute to the findings and will add to the strength of this, and subsequent studies.

### Challenges

Undertaking a study of a community-delivered service by one relatively small care provider organisation presents a number of logistical challenges for the researchers when seeking to organise interviews and focus groups. Ethnographic studies are time-consuming to undertake but it is felt that the richness of data obtained makes this an appropriate approach.

## Conclusion

Through participant observation and targeted data collection through interviews, focus groups and document review, this study provides a rich profile of the way a community-based palliative care service is delivered from an information flow, organisational and cultural perspective. It examines the views of a broad spectrum of Hospice staff regarding the current care delivery approach, and their perceptions of the impact a technology-enabled service could have upon the patients and families they support, and upon themselves personally, the community-based care program, and for the Hospice. The project will develop and test programme theories to describe in detail the linkages between current working practices and organisational and individual outcomes, and proposed theories of change associated with ICT implementation. This will provide both evidential and interpretive baselines against which future evaluation findings can be compared, and from which underlying theories can be further refined.

## Abbreviations

CCG: Clinical Commissioning Group
CLAHRC-YH: Collaboration for Leadership in Applied Health Research and Care for Yorkshire and Humber
EnComPaSS: Enhanced Community Palliative support Service
ICT: Information and communications technology
NIHR: National Institute for Health Research
PROMs: Patient reported outcome measures
